# Intake and Biomarkers of Folate and Risk of Cancer Morbidity in Older Adults, NHANES 1999-2002 with Medicare Linkage

**DOI:** 10.1371/journal.pone.0148697

**Published:** 2016-02-10

**Authors:** Jing Hu, WenYen Juan, Nadine R. Sahyoun

**Affiliations:** 1 University of Maryland, Department of Nutrition and Food Science, College Park, Maryland, United States of America; 2 Office of Nutrition, Labeling and Dietary Supplements, Center for Food Safety and Applied Nutrition, United States Food and Drug Administration, College Park, Maryland, United States of America; RWTH Aachen, GERMANY

## Abstract

**Background:**

After the 1998 mandatory folic acid fortification of enriched cereal-grain products in the U.S., safety concerns were raised that excess consumption of folic acid and high blood folate biomarkers detected in adults may increase the risk of certain types of cancer.

**Methods:**

Baseline data from about 1400 participants in the National Health and Nutrition Examination Survey (NHANES) 1999–2002, aged ≥ 57 years were linked to Medicare and mortality files through December 31, 2007. Using cox proportional hazards regression models, we assessed associations between dietary folate equivalents, folate biomarkers, the presence of unmetabolized folic acid and, overall cancer incidence.

**Results:**

With 8,114 person-years of follow-up (median follow-up, 6.3 years), about 125 cancer cases were identified. After adjusting for confounders, the hazard ratios of the highest quartile versus the second quartile of RBC folate and dietary folate equivalents were 0.54 (95% CI: 0.31–0.93) and 0.54 (95% CI: 0.30–0.95), respectively. Additionally, serum and RBC folate as continuous variables were inversely and significantly associated with cancer incidence (p<0.01). No significant associations were observed between the presence of unmetabolized folic acid, intake of naturally-occurring food folate or folic acid separately, and cancer incidence.

**Conclusions:**

High total folate intake and biomarkers in older adults appear to be protective against cancer in post-folic acid fortification years. This study does not show a negative impact of current level of folic acid fortification on cancer risk. As this is one of the few studies to examine the association between unmetabolized folic acid and cancer outcome, a study including a larger nationwide representative sample of the U.S. population is needed.

## Introduction

Folate is the generic term used for a group of water-soluble vitamins that is both naturally occurring in food and its synthetic form, folic acid, which is added to dietary supplements and to fortified food. Folate is essential for numerous bodily functions. It serves as acceptors or donors of one carbon moieties and are needed in nucleotide synthesis. During digestion, folic acid is reduced to biologically active forms that are identical to those from naturally occurring food folate. Since the human body does not have the ability to synthesize folate, insufficient folate consumption can lead to folate deficiency [1]. This deficiency can result in many adverse health outcomes. The most notable one is neural tube defects (NTDs) in developing embryos. To reduce the incidence of NTDs, the U.S. government mandated folic acid fortification of enriched cereal-grain products in 1998, which supplies on average an additional 100–200 μg folic acid per person per day based on the average American dietary pattern [2]. This folic acid fortification has significantly reduced the frequency of NTDs by 25–30% [3] and has also improved the folate nutritional status of the general U.S. population. Mean folate biomarkers such as serum folate and red blood cell (RBC) folate concentrations increased by approximately 100% and 55%, respectively, in post-fortification (1999–2010) compared to pre-fortification (1988–1994) years [4]. Naturally occurring folate is found predominantly in green leafy vegetables, citrus fruits and legumes, including different types of beans. Vegetables used to be the main contributors of folate to the U.S. diet, however, since folic acid fortification, the category "bread, rolls, and crackers" became the single largest contributor of folate [5].

Though folic acid fortification policy has been successful in reducing the incidence of NTDs and improving folate status of the U.S. population, it also raises safety concerns that high total folate intake may have adverse health effects such as increasing certain types of cancer incidence. It is hypothesized that high intake of folate could promote the progression of pre-existing cancer by providing substrates for DNA replication in rapid cell division [6]. Currently, about 11.3% of the U.S. population exceeds the Tolerable Upper Intake Level (UL) of 1 mg/day, as an outcome of consumption of folic acid fortified foods and dietary supplement use [7]. Moreover, unmetabolized folic acid (UMFA) has become widespread in the systemic circulation of the U.S. population and may be of public health concern. It is reported to be prevalent in approximately 38% of U.S. adults aged ≥ 60 years, according to data from the National Health and Nutrition Examination Survey (NHANES) 2001–2002 [8]. It is speculated that UMFA may be involved in the pathogenesis of cancer by disturbing cellular folate uptake and normal intracellular folate metabolism [9–11]. However, few studies examined the relationship between UMFA and cancer risk and the results are inconclusive.

We conducted this study to examine the associations of folate intake, folate biomarkers, and the presence of UMFA with overall cancer incidence among adults aged ≥ 57 years who participated in NHANES 1999–2002, using the linked Medicare and mortality data.

## Materials and Methods

Baseline data for this study were obtained from the publicly released NHANES 1999–2002. NHANES is a nationally representative survey of the health and nutritional status of the non-institutionalized U.S. population, and it uses a complex, multistage, probability-sampling design. Each NHANES participant undergoes a household interview and a physical examination in a Mobile Examination Center (MEC) [12,13].

The data of NHANES 1999–2002 participants which are de-identified and anonymized were subsequently linked longitudinally to Medicare and mortality data using the NHANES assigned sequence number ([14, 15]. Medicare claims record and death records are restricted-use to protect the confidentiality of survey respondents. Therefore, analysis was conducted in-person at the National Center for Health Statistics (NCHS) Research Data Center after special permission was obtained from the NCHS Ethics Review Board. The University of Maryland, College Park (UMCP) Institutional Review Board also approved the study. More details about NHANES and its methods can be found elsewhere [16]

### Medicare claims and mortality data linkage

Data of NHANES 1999–2002 participants were linked to Medicare enrollment and claim records collected by the Centers for Medicare and Medicaid Services (CMS). The Medicare Chronic Condition Summary File is a summary of clinical information extracted from the NCHS-CMS linked data, which includes the date of first occurrence for 21 chronic conditions, including colorectal, breast, prostate, lung and endometrial cancers from 1999 to 2007. Cancer cases were identified using NCHS designed algorithms of disease codes from Medicare claim records. More details about algorithms of codes can be found in Appendix B of the NCHS-CMS Medicare Chronic Condition Summary File Data Dictionary [17].

Data of NHANES 1999–2002 participants were also linked to the National Death Index (NDI) through December 31, 2006. The date of death, underlying and multiple causes of death in ICD-10 were recorded in the Linked Mortality Restricted-use File [14]. More details about the linked Medicare and Mortality files can be found elsewhere [14, 15]. The Medicare Chronic Condition Summary File information is only available for successfully matched NCHS survey participants who were alive between 2005 and 2007 [15]. If participants were not alive in 2005, data on the incidence of cancer of these participants were obtained from the NHANES Linked Mortality Restricted-use File.

### Ascertainment of cancer cases

Cancer cases were identified from the NHANES Linked Medicare or mortality files. For participants whose data were linked to the Medicare Chronic Condition Summary File and who were alive between 2005 to 2007, cancer cases and the date of first cancer occurrence between 1999 and 2007 were identified from the Summary File [17]. For participants who died between 1999 and 2005, Medicare claims data were not included in the Medicare Chronic Condition Summary File and cancer cases were identified from the Linked Mortality Restricted-use File. Underlying or multiple cause of death from colorectal, breast, prostate, lung and endometrial cancer were identified and the date of death was used as the date of cancer incident.

### Study population

We used data of NHANES 1999–2002 participants aged 57 years and over (n = 3997) to capture all individuals who would potentially reach the age of Medicare eligibility by 2005 to 2007 (Medicare has age-based entitlement at 65 years of age). To protect the confidentiality of survey respondents, NCHS does not release age of respondents over 85 years, so age was capped at 85. There were 3192 NHANES 1999–2002 participants whose data were linked to the Medicare data and 130 participants who had a year of death between 1999 and 2005. Data of Medicare beneficiaries who were enrolled in managed care plans were excluded from the analysis (n = 1034), because CMS generally does not receive claims data for these beneficiaries. Data were also excluded if individuals had renal dysfunction (serum creatinine > 131 μmol/L in men and > 115 μmol/L in women, n = 84), liver disease (serum alanine aminotransferase > 40 units/L, n = 182) or cancer (other than skin cancer, n = 213) at baseline examination. We also excluded data of participants who had missing information on dietary folate or blood folate biomarkers (n = 275) and on weight or height measures (n = 109). The merged analytical sample was comprised of 1425 persons, which included 1384 individuals whose data were linked to the Medicare Chronic Condition Summary File and 41 individuals with data linked to the Mortality File. In the final merged analytical sample, there were missing data on covariates used in the analysis resulting in a sample of 1387 (n = 125 deceased) for total dietary folate, 1384 for serum folate (n = 123 deceased) and 1402 (n = 124 deceased) for RBC folate.

### Assessment of diet and supplement use

Energy and nutrient intakes including naturally occurring dietary folate and folic acid from fortified foods were estimated from a 24-hour dietary recall, which was administered to each participant by NHANES trained dietary interviewers in the MEC. Data on dietary supplements were collected through the NHANES Dietary Supplement Questionnaire at the household interview. Participants were asked a series of questions on vitamin or mineral supplement use during the past 30 days. Detailed information on the frequency of consumption, duration of use, and dosage was collected for each reported dietary supplement. The average daily folic acid intake from dietary supplements and from foods fortified with folic acid was summed to reflect total daily folic acid exposure. In our study, dietary folate equivalents (DFEs) were used as the measure of total folate intake to account for the difference in the bioavailability of naturally occurring food folate and folic acid. DFEs were calculated using the following equation: DFEs (μg) = food folate (μg) + 1.7 × folic acid from fortified foods or supplements (μg) [18].

### Biochemical measurements

RBC folate and serum folate concentrations were measured with the Bio-Rad Laboratories "Quantaphase II Folate/vitamin B12" radioassay from BioRad, Hercules, CA [19]. UMFA concentrations were determined in NHANES 1999–2002 only for participants aged 60 years and over by using a revised affinity/ HPLC method with electrochemical (coulometric) detection [20]. The lower limit of detection for UMFA was 0.18 nmol/L and values below the level of detection were set to zero. Serum creatinine was measured based on the Jaffe reaction. Serum alanine aminotransferase (ALT) was measured using an enzymatic rate method. Complete details and documentation for each of these methods are described elsewhere [19, 20].

### Demographic characteristics and lifestyle variables

Demographic characteristics and certain lifestyle behaviors were collected using interviewer-administered questionnaires and conducted in participants’ home. Individuals were classified by race/ethnicity as non-Hispanic white, non-Hispanic black and others (Mexican American, other Hispanic and other race/ethnicity). Educational attainment was categorized as less than high school, high school graduate (received a high school or high school equivalency diploma), and greater than high school. Physical activity level was self-reported as sedentary, light and, moderate/high intensity and cigarette smoking status was categorized as never, former, and current smokers. A never smoker was defined as a participant who reported not having smoked 100 or more cigarettes during his/her lifetime; a former smoker smoked 100 or more cigarettes but was not smoking at the time of the interview; and a current smoker smoked 100 or more cigarettes and was smoking at the time of interview. Heights and weights were measured at the MEC and body mass index (BMI) was calculated as weight in kilograms divided by height in meters squared (kg/m^2^). Alcohol consumption was calculated as grams per day from the 24-hour dietary recall collected at the MEC

### Statistical analyses

Baseline data from NHANES 1999–2002 (publicly available), incidence of cancer obtained from the Medicare Chronic Condition Summary File, and mortality data obtained from the Linked Mortality Restricted-use File were merged into a single file at the NCHS Research Data Center computer lab. Analyses were performed using SAS (version 9.2; SAS Institute Inc., Cary, NC) and p < 0.05 was considered statistically significant.

Cox proportional hazards regression models were used to examine hazard ratios (HR) and 95% confidence intervals (95% CI) for incidence of cancer by quartiles of folate intake and folate biomarkers. The second quartile, which includes the Recommended Dietary Allowance (RDA) for folate for adults aged 19 years and older, was used as the reference group in the regression models. Models were also developed to examine the folate variables in their continuous forms. UMFA measurements were categorized into detectable UMFA (UMFA+) and undetectable UMFA (UMFA-) and was treated as a categorical variable in the regression model. The follow-up period of participants in survival analyses was estimated from the time of baseline data collection to the endpoint (the earliest occurrence of any cancer: lung, prostate, breast, colorectal or endometrial cancer) or end of study. People who were alive or died of other causes were censored at the end of the follow-up period (December 31, 2007) or at the date of death.

ANOVA for continuous variables and Chi-squared tests for categorical variables were used to examine associations between quartiles of folate biomarkers (serum folate and RBC folate) or dietary folate intake (naturally occurring food folate, folic acid, and DFE) and demographic and lifestyle characteristics of survey participants to assess for potential confounders. Cox proportional hazards regression models were adjusted for the demographic confounders; age, gender, race/ethnicity, and educational attainment and for the lifestyle variables; smoking, BMI, physical activity and alcohol intake that were associated with the dependent variables. Also, total energy intake, which is associated with DFE was added to the DFE and cancer regression models. Serum folate, RBC folate, food folate, folic acid and DFE were logarithmically transformed, because these variables were not normally distributed. The proportionality assumption in the Cox model states that there is a constant relationship between the dependent variables and explanatory variables so that the effect of any predictor variable is constant over time. This was tested by creating the interaction term (predictor*log time) and the p-value for Chi-square was checked. None of the interaction terms were significant and, therefore, there were no violations to this rule.

## Results

With 8,114 person-years of follow-up (median follow-up, 6.3 years), over 125 cancer cases, depending on model, were identified from the Medicare data; fewer than ten cancer cases were identified from the mortality data based on underlying or multiple causes of death. The characteristics of the cohort by quartiles of RBC folate and DFE are summarized in Table 1. RBC folate concentrations tended to be higher among non-Hispanic white women with higher educational attainment, never smokers, and users of vitamin and mineral supplements. Participants who had higher intake of dietary folate equivalents tended to be non-Hispanic white men, with higher educational attainment, more physically active, former smokers, and users of vitamin and mineral supplements.

**Table 1:** Characteristics of NHANES 1999–2002 participants by quartiles (Q) of red blood cell (RBC) folate and dietary folate equivalents (DFE)^1^.

	RBC folate (ng/ml)				DFE (μg/d)			
	Q1	Q2	Q3	Q4	Q1	Q2	Q3	Q4
	<237.8	237.8–<318.0	318.0–<422.0	≥422.0	<291.6	291.6–<467.0	467.0–<836.4	≥836.4
Characteristics^2^								
n	341	361	350	350	345	353	351	338
Days of follow up	2193 ± 39	2096 ± 42	2179 ± 39	2187 ± 40	2162 ± 45	2193 ± 41	2201 ± 40	2091 ± 33
Age (years)	68.6 ± 0.4	69.2 ± 0.4	70.8 ± 0.5	71.2 ± 0.5	70.2 ± 0.5	69.7 ± 0.4	69.9 ± 0.5	69.6 ± 0.4
Gender, men (%)	59.2	55.1	50.6	44.9	44.3	54.1	57.6	55.6
Race/ethnicity (%)								
Non-Hispanic white	41.4	55.2	69.1	75.4	49.3	56.9	63.5	71.1
Non-Hispanic black	29.6	15.4	8.9	5.3	21.1	16.2	12.0	10.1
Other race	29.0	29.4	22.1	19.3	29.6	26.9	24.5	18.9
Education level (%)								
< High school	54.1	48.7	33.2	32.5	55.1	45.6	36.5	31.1
High school	18.9	20.5	27.2	23.4	19.1	23.8	22.5	25.7
> High school	26.9	30.8	39.5	44.2	25.8	30.6	41.0	43.2
Smoking (%)								
Current	24.0	14.9	12.3	10.5	21.2	18.1	10.3	11.8
Former	34.6	40.6	36.4	36.8	33.9	35.4	40.7	41.7
Never	41.4	44.5	51.3	52.6	44.9	46.5	49.0	46.5
Physical activity (%)								
Sedentary	29.0	31.1	25.8	29.5	35.5	30.3	27.9	24.9
Light	58.3	55.7	59.3	55.6	54.5	56.4	57.3	57.1
Moderate/high	12.7	13.2	14.9	14.9	9.7	13.1	15.0	18.1
Alcohol intake (gm/d)	6.5 ± 1.2	6.5 ± 1.1	4.0 ± 0.7	4.5 ± 0.9	4.2 ± 0.6	5.9 ± 1.1	6.1 ± 1.1	5.2 ± 0.9
BMI (kg/m^2^)	27.6 ± 0.3	28.4 ± 0.3	28.1 ± 0.3	28.0 ± 0.3	28.3 ± 0.3	28.1 ± 0.3	28.0 ± 0.3	27.7 ± 0.3
Total energy intake (kcal/d)	1730 ± 45	1797 ± 42	1807 ± 40	1760 ± 41	1186 ± 24	1725 ± 31	2044 ± 40	2111 ± 47
Folic acid supplement, users (%)	7.1	19.3	13.8	62.6	13.8	17.7	27.4	74.6

^1^ Frequencies do not add up to 100% because of rounding and missing values.

^2^ Mean ± SE for continuous variables, and percentages for categorical variables presented by quartiles of RBC folate and intake of DFE.

Results from the Cox regression analysis showed that individuals whose RBC folate levels were in the fourth quartile (≥ 422.0 ng/ml) had a significantly lower risk of cancer incidence compared to the reference group (237.8 to <318.0 ng/ml); the adjusted HR was 0.54 (95% CI: 0.31–0.93). This was also true for individuals whose DFE intakes were in the fourth quartile (≥836.4 μg/d) compared to the reference category (291.6 to <467 μg/d); the adjusted HR was 0.54 (95% CI: 0.30–0.95). However, the association between the highest serum folate category and risk of cancer incidence only approached significance (HR: 0.59, 95% CI: 0.33–1.05). (Table 2). When folate biomarkers and DFE were examined as continuous variables, results showed an inverse significant association between both RBC and serum folate and risk of incident cancer but no associations with DFE (Table 3). There were no associations observed between naturally occurring food folate, folic acid or the presence of UMFA and risk of cancer incidence (data not shown).

**Table 2:** Hazard ratios (HR) of overall cancer and 95% confidence intervals (95% CI) by quartiles (Q) of red blood cell (RBC) folate, serum folate, and dietary folate equivalents (DFE) ^1^^,^^2^, NHANES 1999–2002.

	Q1	Q2	Q3	Q4
Red blood cell folate (ng/ml)	<237.8	237.8–**<**318.0	318.0–**<**422.0	≥422.0
Sample size, n	341	361	350	350
All-cancer cases, n (%)	38 (11.1)	39 (10.3)	27 (7.7)	20 (5.7)
HR, adjusted (95% CI)	0.98 (0.61–1.57)	1.0	0.68 (0.41–1.14)	0.54 (0.31–0.93)
Serum folate (ng/ml)	<10.7	10.7–**<**15.6	15.6–**<**22.9	≥22.9
Sample size, n	334	350	355	345
All-cancer cases n (%)	42 (12.6)	32 (9.1)	30 (8.4)	19 (5.5)
HR, adjusted (95% CI)	1.44 (0.90–2.31)	1.0	0.88 (0.53–1.47)	0.59 (0.33–1.05)
DFE (μg/d)	<291.6	291.6–<467.0	467.0–**<**836.4	≥836.4
Sample size, n	345	353	351	338
All-cancer cases n (%)	31 (9.0)	38 (10.8)	38 (10.8)	18 (5.3)
HR adjusted, (95% CI)	0.85 (0.51–1.41)	1.0	0.95 (0.60–1.52)	0.54 (0.30–0.95)

^**1**^ Tests were performed using Cox proportional hazards regression models that included log-transformed RBC folate, serum folate or DFE

^**2**^ Models were adjusted for age, gender, race/ethnicity, educational attainment, smoking status, alcohol intake, physical activity, and BMI. The DFE model was additionally adjusted for total energy intake.

**Table 3:** Hazard ratios of overall cancer incidence and 95% confidence intervals (95% CI) by continuous levels of red blood cell (RBC) folate, serum folate, and dietary folate equivalents (DFE) ^1^, NHANES 1999–2002.

	Hazard ratio (95% CI)^2^	Coefficient	P
RBC folate (ng/ml)	0.57 (0.40–0.81)	-0.57	< 0.01
Serum folate (ng/ml)	0.51(0.32–0.80)	-0.68	< 0.01
DFE (μg/d)	0.81(0.60–1.08)	-0.22	0.15

^**1**^ Tests were performed using Cox proportional hazards regression models that included log-transformed RBC folate, serum folate or DFE as independent continuous variables.

^2^ Models were adjusted for age, gender, race/ethnicity, educational attainment, smoking status, alcohol intake, physical activity, and body mass index. The DFE model was additionally adjusted for total energy intake.

## Discussion

This study shows inverse significant associations between total folate intake, folate biomarkers and overall cancer risk. The highest quartile of RBC folate was significantly inversely associated with overall cancer incidence and so was the highest quartile of DFE compared to the reference category. Also, RBC folate and serum folate as continuous variables were inversely associated with cancer risk.

Folate is an essential vitamin in the maintenance of normal DNA function. It is a cofactor in DNA synthesis, repair and methylation. Folate deficiency is considered a risk factor for cancer. Inadequate folate may increase cancer risk by disturbing the synthesis of thymidylate and purines [21, 22], which may cause uracil misincorporation into DNA. Uracil misincorporation may destabilize DNA and increase risk of malignancy. A deficiency in folate also affects methylation of DNA, which in turn influences gene expression and triggers carcinogenesis [22]. It’s been suggested that the mechanisms through which folate influences DNA function may result in dual effects of folate on cancer, whereby low folate concentrations could trigger cancer initiation, while excessive folate intake could promote more rapid cancer progression following onset by providing DNA synthesis substrates [23].

In our study, we did not observe a higher risk of cancer in individuals in the lowest DFE quartile (< 292 μg DFE/day) compared to the reference group that included the RDA value for adults aged 19 and older (400 μg DFE/day). Several epidemiological studies conducted in pre-folic acid fortification years in the U.S. suggest that low folate intake (< 200 μg/day) is associated with an increased risk of cancer incidence [24, 25]. Due to the mandatory folic acid fortification policy only about 10% of individuals aged 57 years and older had total folate intake less than 200 μg per day in our study. The small number of individuals with lower intake of folate may not have provided sufficient statistical power to detect significant associations.

However, we did observe that individuals with DFE intake and RBC folate in the highest quartile had lower cancer risk. Also, there was an inverse association between continuous RBC folate, serum folate and risk of all-cancer incidence. These findings do not support the hypothesis that fortification of enriched cereal-grain products with folic acid increase the incidence of cancer. Several studies have reported positive associations between high folate intake levels and cancer risk. In two ecological studies, the increase in intake of folate due to folic acid fortification was found to parallel an increase in the incidence of colorectal cancer in the U.S., Canada [26] and Chile [27]. In a clinical trial, administration of folic acid supplementation (1000 μg/d) in patients with a history of adenoma was found to accelerate the growth of adenomas and increase the risk of cancer [28]. Additionally, higher intake of folate from either supplemental folic acid (≥ 400 μg/d) or dietary folate (≥ 312 μg/d) was associated with an increased risk of postmenopausal breast cancer [29, 30]. On the other hand, a recent meta-analysis that pooled data of approximately 50,000 participants in randomized clinical trials found that folic acid supplementation did not significantly increase or decrease the incidence of site-specific cancer during the first 5 years of treatment. The doses used in these trials were higher than the average amounts consumed from folic acid fortification of enriched cereal-grain products [31].

Finally, we did not find UMFA to be significantly associated with cancer risk. Folic acid, the synthetic form of folate, needs to be reduced to its biologically active form (tetrahydrofolate) by the enzyme dihydrofolate reductase (DHFR) before taking part in intracellular reactions. High intake of folic acid could saturate the enzyme and result in the buildup of UMFA in circulation [32]. In post-folic acid fortification years, UMFA has been reported to be prevalent in about 38% of U.S. adults aged ≥ 60 years due to high consumption of folic acid from dietary supplements and fortified foods [8]. Little is known about possible intracellular effects of UMFA. It is hypothesized that UMFA could interrupt normal folate metabolism through several mechanisms, including inhibiting folate-dependent enzymes and interfering with DNA synthesis and methylenetetrahydrofolate reductase (MTHFR) [11, 33, 34]. However, to date few studies examined the association between UMFA and health outcomes. One study found that high blood UMFA concentrations were associated with decreased natural killer cell cytotoxicity among postmenopausal women [11]. In another study, researchers examined the association between UMFA collected pre-folate fortification of food and colorectal cancer. The results were inconclusive [35]. In our study the absence of a relationship between UMFA and the incidence of cancer may be due to insufficient power to detect a significant association, as UMFA was only measured for participants aged 60 years and over in NHANES 1999–2002. Additionally, because not all participants had detected UMFA in the blood, we examined the association between the presence versus absence of UMFA rather than the amount of UMFA and risk of cancer incidence. Further study is needed in larger population samples to examine a potential dose-response association between UMFA and cancer risk.

A limitation of this study is that cancer cases were identified using algorithms of disease codes from medical claims records. Medicare claims data were collected for billing purposes and not for epidemiological study, and, therefore, may not reflect precise disease occurrence [36, 37]. However, studies that examined this methodology showed that Medicare claims data have reasonably high sensitivity for the detection of cancer in older adults [38, 39]. Also, the strength of using Medicare claims data is that they are not subject to recall bias.

Another limitation of our study is that we used overall cancer morbidity as the health outcome because the number of site-specific cancer cases was too small. Folate may have different effects on the etiologies of different cancers. Additionally, the date of death from cancer was used as the date of cancer incidence for individuals who died between 1999 and 2005. The date of first occurrence was not available for participants who died before 2005. As fewer than ten cancer cases were identified through the mortality files, this would not have had much impact on the results. Finally, due to our exclusion criteria, the study sample may not be nationally representative of the U.S. population.

In conclusion, our findings indicate that DFE and folate biomarkers are associated with lower risk of overall cancer incidence and suggest that folate may have a protective role against cancer even at post-folic acid fortification levels. UMFA detected in serum was not associated with cancer risk. Studies with larger nationally representative samples and similar dietary folate and biomarker levels are needed to confirm these findings.
